# Bacterial community composition of biofilms in milking machines of two dairy farms assessed by a combination of culture-dependent and –independent methods

**DOI:** 10.1371/journal.pone.0222238

**Published:** 2019-09-11

**Authors:** Mareike Weber, Janine Liedtke, Susanne Plattes, André Lipski

**Affiliations:** 1 Department of Food Microbiology and Hygiene, Institution of Nutrition and Food Science, Rheinische Friedrich-Wilhelms-University, Bonn, North-Rhine-Westfalia, Germany; 2 CIDRe, Rheinische Friedrich-Wilhelms-University Bonn, Bonn, North-Rhine-Westfalia, Germany; Universidad Nacional Autonoma de Mexico Facultad de Quimica, MEXICO

## Abstract

Dairy biofilms as a source of contamination of milk and its products are of great concern in the dairy industry. For a reliable risk assessment, knowledge about the microbial community composition of biofilms in the milking systems of dairy farms must be improved. In this work, swab samples of milking machine biofilms of two dairy farms were investigated by a combination of culture-dependent and -independent methods. Spots in the milking system with enhanced microbial colonization were identified by quantification on selective and non-selective media. In addition, stainless steel coupons were placed into the piping system of a milking machine, removed after several milking intervals, and investigated for colonization by cultivation and culture-independently. Isolates were differentiated and identified by a combination of chemotaxonomical methods and 16S rRNA sequencing. The culture-independent approach involved treatment of the samples with the viability dye propidium monoazide prior to direct DNA-extraction by enzymatic cell lysis and cloning to exclude bias from dead biomass. The milking equipment retainers and the outlet of the milk bulk tank were identified as highly colonized spots on both farms. A high bacterial diversity was detected covering the phyla *Actinobacteria*, *Bacteroidetes*, *Firmicutes*, and *Proteobacteria*. Presence of biofilms was demonstrated on several materials including stainless steel and plastic, which are frequently used in milking machines, but also in dairy processing plants. Growth of mainly Gram-positive bacteria with high percentages of the phylum *Actinobacteria* was detected on the stainless steel coupons after exposition in the milking system for two to three days. Knowledge about the heterogenic microbial load on different parts of the milking machines and the stainless steel coupons will help to identify primary colonizers of the milking system, to assess the risk potential of biofilms for raw milk, to improve sanitation processes and to identify parts of the milking machine, which should be improved by hygienic design.

## Introduction

Biofilms are accumulations of microbial cells attached to surfaces and embedded in self-produced matrices [1]. Attachment of cells and subsequent biofilm formation can be influenced by surface material, roughness, and hydrophobicity, which can in turn be affected by surface conditioning with food ingredients [2, 3].

Bacterial biofilms are a major problem in the food industry. Once attached, biofilm cells can withstand unfavorable environmental conditions, such as nutrient depletion or treatment with antimicrobial substances [4, 5]. Thus, it is difficult or impossible to remove mature biofilms by standard cleaning and sanitation processes [6].

For the dairy industry biofilm formation is a fundamental problem, because raw milk contains a wide variety of microorganisms and dairy products are susceptible to microbial spoilage. Even if primary biofilm formers are neither spoilage organisms nor pathogens, established biofilms can become a habitat for harmful organisms like *Listeria monocytogenes* [7]. Biofilms on surfaces with contact to pasteurized products pose a threat to human health and product quality [8, 9]. Additionally, the secretion of heat-resistant spoilage enzymes such as proteases and lipases by biofilm inhabitants into the raw milk can lead to reduced shelf-life of UHT milk [3, 10].

The bacterial community composition of raw milk has been described in detail by culture-dependent [11, 12] and–independent methods [13, 14], as well as by a combination of both [15–18]. For direct molecular methods, the effectiveness of DNA-extraction and the application of viable-PCR (vPCR) are crucial for the completeness and comparability of the depicted bacterial community composition [18].

The microbiota of udders and of the stable environment have already been described as sources for raw milk contamination [12, 17]. Dairy biofilms have been analyzed in sprinklers from dairy farm cooling systems [19], raw milk pipelines on dairy farms [20], raw milk cooling tanks [21], raw milk road tankers [10] and in dairy processing plants [8, 22, 23] so far. Many studies focus on the detection of pathogens, like *Listeria monocytogenes* [24], or on other prominent milk associated bacterial genera [25].

Biofilms in milking machines on dairy farms may affect udder health as well as the raw milk microbial load and microbiota. The spread of biofilm forming bacteria from raw milk to milk tankers and dairy processing plants affects the composition of biofilms in these areas.

To our knowledge, there are no data available about the microbial community composition of biofilms in milking systems detected by a combination of culture-dependent and -independent methods on different dairy farms. Those data are essential to assess the contribution of the milking system itself to raw milk contamination in addition to teat microbiota or other cow-associated microorganisms. The comparative investigation of different dairy farms can reveal typical bacterial genera present in milking machine biofilms and detect critical spots in the machine milking process with increased biofilm formation. Moreover, the recolonization dynamics of milking systems after cleaning and sanitation procedures are an important aspect for hygienic evaluation of the milking system operation. Therefore we included experiments with stainless steel coupons placed into the piping system of an operating milking system for several days to track the process of biofilm formation. Tracking biofilm formation on stainless steel coupons will help to identify bacterial genera initiating biofilm formation on stainless steel in the dairy environment under realistic conditions. The characterization of biofilms in milking machines as the starting point of dairy production can help to optimize cleaning and sanitation processes.

The aim of the present work was comparing the bacterial community composition of biofilms in the milking machines of two different dairy farms and tracking biofilm formation on stainless steel coupons placed into one milking machine during ongoing operation. To increase the detected bacterial diversity, a parallel approach of culture-dependent and -independent methods with viable-PCR (vPCR) was applied.

## Materials & methods

### Biofilm sampling

#### Characterization of the investigated dairy farms

Farm 1 (F1) is a research farm of the University of Bonn (Königswinter, Germany), where the raw milk of 60 German Holstein cows is collected twice daily. The milking machine and the bulk tank (Kryos 6BII, WestfaliaSurge Japy SAS, Saint Apollinaire, France) are cleaned in a cleaning in place (CIP) regime by alkaline (Circo Super AFM) or acidic treatment (Circo Super SFM) (GEA Farm Technologies, Bönen, Germany). The active compounds of the alkaline treatment are sodium hypochloride and sodium hydroxide, while the acidic treatment combines phosphoric acid and nitric acid. Alkaline and acidic treatments are applied alternating between each milking interval. The detergents are diluted automatically, resulting in final concentrations of 0.4%, as recommended by the manufacturer. Each treatment is preceded and followed by a water rinse.

Farm 2 (F2) is an owner-managed facility in Schlausenbach (Germany) with 18 cows milked twice daily. The milking machine and the bulk tank (Westfalia Systemat, WestfaliaSurge Japy SAS, Saint Apollinaire, France) are cleaned with an alkaline agent (Eifelrein A, Arla Foods Deutschland GmbH, Pronsfeld, Germany) with concentrations of 0.2% at 40°C for 30 min (milking machine) and 0.3% at 30°C for 15 min (bulk tank), respectively. Each cleaning cycle is preceded by a five-minute water rinse and followed by a 10 min water rinse.

We confirm that all authorities issued the permission for the field studies described. Owner of farm 1 is the Bau- und Liegenschaftsbetrieb (BLB) NRW (building and real estate management of the state North Rhine-Westphalia). The owner granted permission for this study. Owner of farm 2 is Susanne Plattes. She is a co-author of this study and permission was granted by her.

#### Swab sampling

**Fig 1:**
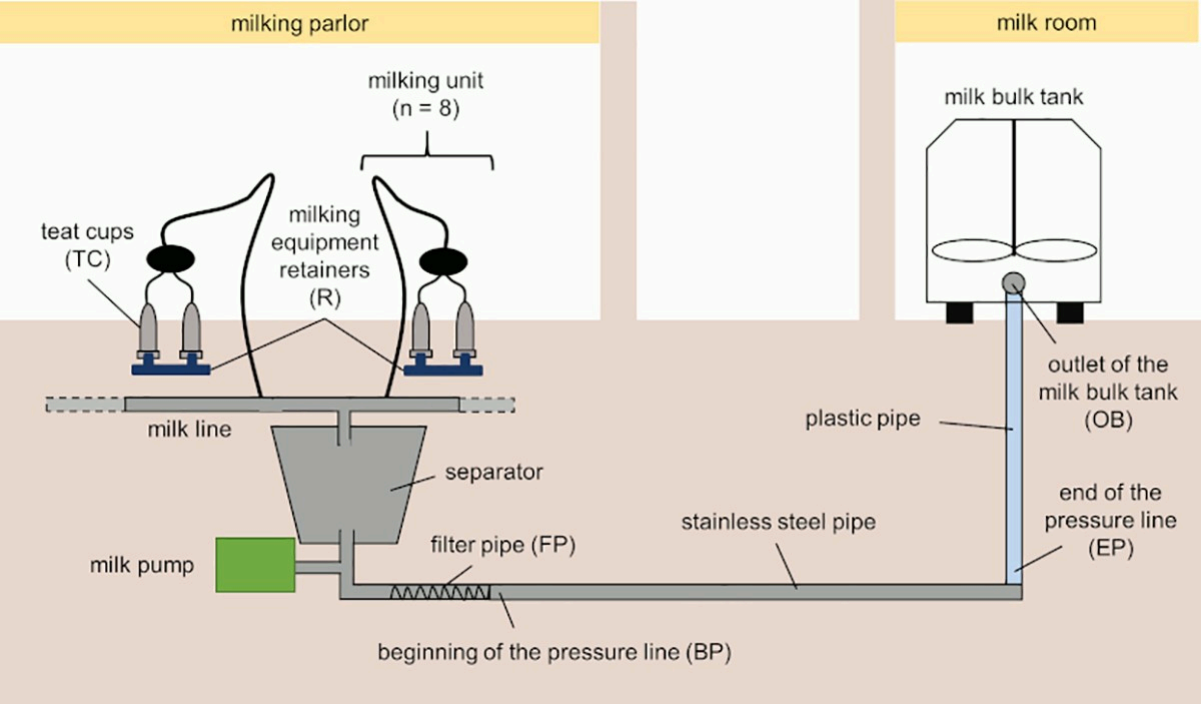
Schematic diagram of the milking machine of farm 1. The proximity to the udders decreases from the teat cups to the outlet of the milk bulk tank.

Swab samples of biofilms were taken from the milking machines of the two dairy farms about 4 h after the cleaning and sanitation process. Samples were taken from the teat cups (**TC**), the milking equipment retainers (**R**), the stainless steel pipe containing the in-line milk filter (**FP**), a stainless steel pipe at the beginning of the pressure line (**BP**), a plastic pipe at the end of the pressure line (**EP**) and the outlet of the bulk tank (**OB**). Fig 1 shows a schematic diagram of the milking machine of farm 1.

Samples of farm 1 were taken in March and August 2014 and in June 2015. The 2014 samples were investigated by the culture-dependent method, while the 2015 samples of farm 1 and the samples of farm 2, taken in October 2014, were investigated culture-dependently and -independently in parallel approaches.

#### Stainless steel coupons

Stainless steel coupons (7.5 x 2.5 cm) (V2A, 1.4301, X5CrNi18-10) were cleaned by incubation in acetone for at least 3 h, rinsed with distilled water and sterilized by autoclaving. Sterilized and cleaned coupons were placed into the spiral holding the filter at the beginning of the pressure line in the milking machine of farm 1. They remained within the spiral during ongoing operation of the milking machine (including milking and CIP-processes) until they were removed for investigation. The removal took place at least 4 h after the last CIP-process so that surviving cells could recover in order to be detected by cultivation. During three samplings in November 2015, October 2016 and February 2017, the coupons remained in the milking machine for 48 h, which equals four milking intervals as well as CIP-procedures. In another sampling in December 2015, the coupons remained in the milking machine for 72 h (six milking intervals/CIP-procedures).

### Enumeration and isolation of bacteria

The swabs were transferred into sterile test tubes containing 10 ml of Ringer’s solution (Merck KGaA, Darmstadt, Germany) and stirred on a vortex mixer for 25 s. Serial dilutions were spread on the following media (all by Merck KGaA, Darmstadt, Germany): trypticase soy agar (TSA) for total microbial count (TMC), violet red bile dextrose agar (VRBD) for the detection of *Enterobacterales*, cetrimide agar (CFC) for *Pseudomonas* spp., Baird Parker agar (BP) for Staphylococci and de Man, Rogosa, and Sharpe agar (MRS) for lactic acid bacteria (LAB). The stainless steel coupons were pressed on the surface of the agar media described above for several seconds. Plates were incubated at 30°C for 72 h. MRS agar was only used for swab samples and stainless steel coupons of farm 1 and was incubated anaerobically. The main colonizers, defined by uniform colony morphology, were selected for isolation from each medium and purified on TSA for subsequent identification.

### Differentiation and identification of isolates

Isolates were characterized according to their colony and cell morphology and differentiated by Gram-staining [26] and KOH lysis test. Fatty acid analysis was conducted for each isolate after growth on TSA for 48 h at 30°C. Fatty acid methyl esters were prepared according to the method of Sasser (1990) [27] and analyzed by GC-MS with a model 7890A (Agilent, Waldbronn, Germany) gas chromatograph equipped with a 5% phenylmethylsilicone capillary column and a model 5975C (Agilent, Waldbronn, Germany) mass spectrometer, as described by Lipski and Altendorf (1997) [28].

The isolates were grouped according to their fatty acid profiles and representative isolates of each group were identified by 16S rRNA gene sequencing, as described previously [18]. They were assigned to a species at ≥ 98.7% sequence similarity [29] with type strain sequences taken from the databases EzTaxon server [30] and GenBank using the Basic local alignment search tool (BLASTN) [31] at the NCBI-website.

### Biofilm formation assays

The biofilm forming potential of the farm 1 isolates was tested in 96-well polystyrene microplates in tryptic soy bouillon (TSB, Merck KGaA, Darmstadt, Germany) according to the method of Kolari *et al*. (2003) [32] with minor modifications. The cell density per well was adjusted to 1 x 10^6^ cfu/ml in 200 μl TSB. After incubation (30°C, 24 h), the wells were emptied and filled with 250 μl of crystal violet solution (4 g/l in 20% methanol). After 5 min of incubation, the plates were emptied, washed three times with distilled water (Nunc Immuno^TM^ Washer, Thermo Scientific, Waltham, USA) and the dye was extracted by addition of 300 μl of ethanol. After 60 min, the absorbance at 550 nm was measured by a microplate reader (Epoch Reader, BioTek, USA). The mean absorption of the blank was subtracted from the absorption of the isolates. The biofilm forming ability of the isolates was propotional to their mean absorption (n = 4) and classified as strong (Abs _550 nm_ ≥ 2.0), moderate (2.0 > Abs _550 nm_ ≥ 0.5), weak (0.5 > Abs _550 nm_ ≥ 0.1) or no biofilm formation (Abs _550 nm_ < 0.1).

### Spoilage potential

Lipolytic activity was analyzed on tributyrin agar (TSA supplemented with 1.0% v/v tributyrin) and proteolytic activity was analyzed on TSA containing 5% (w/v) skim milk powder [33]. Plates were incubated at 30°C for 72 h.

### PMA treatment, DNA extraction and cloning of the 16S rRNA genes

Both sides of a stainless steel coupon were rubbed with swabs wetted in Ringer’s solution. The swabs were transferred into sterile test tubes containing 10 ml of Ringer’s solution and the cells were removed by stirring. 2 ml of the suspensions were treated with propidium monoazide (PMA) [stock solution: 20 mM PMA (Biotium Inc., Hayward, CA) in 20% dimethyl sulfoxide (AppliChem GmbH, Darmstadt, Germany)] at a final concentration of 75 μM. Incubation in the dark and PMA activation by light exposure were conducted as described previously [18].

After centrifugation at 13,000 x g for 10 min, the cell pellet was resuspended in 180 μl of lysis buffer [20 mM Tris HCl (pH 8.2), 2 mM EDTA, and 1.2% Triton X-100] containing lysozyme (20 mg/ml) and lysostaphin (0.15 mg/ml), and 9 μl of mutanolysin was added (stock solution: ≥ 4,000 U in 0.1 M potassium phosphate buffer, pH 6.2) (all chemicals by Sigma Aldrich, Munich, Germany). The mixture was incubated at 37°C for 30 min, then 25 μl of Proteinase K and 200 μl of buffer AL (both Qiagen GmbH, Hilden, Germany) were added. Another incubation step at 70°C for 30 min was followed by addition of 200 μl of ethanol (Merck KGaA, Darmstadt, Germany). Further DNA extraction was performed using the DNeasy Blood & Tissue kit (Qiagen GmbH, Hilden, Germany) according to the manufacturer’s instructions.

The 16S rRNA genes were amplified using the primer pair GM3F and GM4R [34] and the PCR products were purified using the QIaquick PCR purification kit (Qiagen, Hilden, Germany).

The purified PCR products were used to construct clone libraries with the pGEM-T vector system and were transferred into competent *E*. *coli* JM109 cells as specified by the manufacturer (Promega Corp., Madison, WI). Inserts of randomly selected clones were amplified using the primer pair M13F and M13R [35]. PCR products that were confirmed to have the correct length by gel electrophoresis were purified and sequenced as described previously [18]. Prior to identification, all sequences were checked for chimeras by using the Pintail program [36] and chimeric sequences were excluded from further analysis.

### Statistical analysis and construction of phylogenetic trees

Coverage according to Good (1953) [37] was calculated to assess the completeness of the depicted bacterial diversity by the number of sequenced clone inserts. Sequences were assigned to the same operational taxonomic unit (OTU) at ≥ 99% sequence similarity [38]. Shannon-Index (H) and Equitability (E) were calculated for each sample [39].

Representative sequences of each OTU obtained in this study were deposited in the European Nucleotide Archive (ENA) or GenBank under the accession numbers LN717247, LN997865 to LN998002, MK547258 to MK547271, MK547287 to MK547293, LT222224, and LT222225.

## Results

### Bacterial counts

The total microbial count as well as the bacterial counts for presumptive *Enterobacterales*, *Pseudomonas* spp., *Staphylococcus* spp. and lactic acid bacteria are presented in Table 1. The bacterial counts of the teat cups of both farms were lower than the detection limit of 0.4 log_10_ cfu/cm^2^.

**Table 1:** Biofilm bacterial counts on different parts of the milking machines of farm 1 (F1) and farm 2 (F2) and on stainless steel coupons placed into the milking machine of farm 1 for different time intervals.

log_10_ cfu/cm^2^ mean (SD)	Milking equipment retainer (R) plastic		Filter tube (FT) stainless steel		Beginning of the pressure pipe (BP) stainless steel		End of the pressure pipe (EP) plastic		Outlet of the bulk tank (OB) stainless steel		Coupon (C) stainless steel	
	Farm 1 (F1) (n = 9)	Farm 2 (F2) (n = 2)	F1 (n = 3)	F2 (n = 1)	F1 (n = 2)	F2 (n = 1)	F1 (n = 3)	F2 (n = 1)	F1 (n = 3)	F2 (n = 1)	F1, 48 h (n = 3)	F1, 72 h (n = 1)
Total microbial count (TSA)	6.3 (1.1)	4.4 (1.9)	2.8 (0.9)	2.6	2.7 (2.4)	< 0.8	4.2 (0.5)	1.8	3.8 (2.1)	5.5	0.4 (0.4)	1.1
*Enterobacterales* (VRBD)	5.1 (1.1)	2.1 (2.1)	< 0.7	< 0.7	0.9 (0.2)	< 0.8	< 0.7	0.8	2.3 (1.5)	3.8	< dl	< dl
*Pseudomonas* spp. (CFC)	2.8 (1.5)	< 0.6	< 0.7	< 0.7	< 0.7	< 0.8	< 0.7	< 0.8	1.6 (1.5)	2.4	< dl	< dl
*Staphylococcus* spp. (BP)	3.0 (0.7)	< 0.6	1.2 (0.5)	< 0.7	< 0.7	< 0.8	2.3 (1.4)	< 0.8	1.3 (0.8)	< 0.5	< dl	< dl
*Lactic acid bacteria* (MRS)	0.9 (0.5)	nd	1.7 (1.0)	nd	1.9 (1.7)	nd	3.3 (0.6)	nd	1.8 (1.6)	nd	< dl	< dl

Bacterial counts are given as mean log10 cfu/cm^2^ calculated from the indicated number of parallels (n) achieved by pooling of the samples. The standard deviation is given in brackets. nd: not determined; < dl: lower than the detection limit of 1 cfu/18.8 cm^2^; TSA: trypticase soy agar; VRBD: violet red bile glucose agar; CFC: cetrimide agar; BP: baird parker agar; MRS: de Man, Rogosa, and Sharpe agar.

The highest mean TMC of 6.3 log_10_ cfu/cm^2^ was detected on the milking equipment retainers of farm 1, followed by the plastic pipe at the end of the pressure line and the outlet of the milk bulk tank with TMCs about 2 log levels lower. On farm 2, the TMC for the outlet of the milk bulk tank was one log level higher than the mean TMC of the milking equipment retainers. While the TMCs for the filter tubes of both farms were similar, the TMC of the plastic pipe at the end of the pressure line on farm 2 was 2.4 log levels lower than on farm 1.

The high standard deviations on the stainless steel pipe at the beginning of the pressure line and the outlet of the milk bulk tank of farm 1 indicate that the population density ranged from TMCs close to the detection limit to higher TMCs on different sampling occasions. In contrast to this, the standard deviations of all other sampling spots of farm 1 were considerably lower, indicating constant population densities between different samplings within a period of 15 months.

The TMC on the stainless steel coupon exposed in the milking machine for 48 h in October 2016 was lower than the detection limit of 1 cfu/18.8 cm^2^, while the TMCs were 5 and 3 cfu/cm^2^ in November 2015 and February 2017, respectively, resulting in a mean log_10_ cfu/cm^2^ of 0.4. The TMC more than doubled on the coupon with extended exposition time in the milking machine for 72 h.

On both farms, presumptive *Enterobacterales* and *Pseudomonas* spp. were only detected on the milking equipment retainers and the outlet of the milk bulk tank, which were the spots with the highest TMCs. However, no *Pseudomonas* spp. could be detected on the milking equipment retainers of farm 2, and presumptive *Enterobacterales* were only detected on one of the two sampled retainers.

The bacterial counts of presumptive lactic acid bacteria increased from 1.7 ± 1.0 at the filter tube to 3.3 ± 0.6 log_10_ cfu/cm^2^ at the plastic pipe at the end of the pressure line of farm 1. LAB dominated in the pipe system of farm 1 together with presumptive *Staphylococcus* spp. In contrast to this, Staphylococci were not detected in the biofilm samples of the milking machine of farm 2.

### Bacterial community composition

The length of the 16S rRNA gene sequences used for isolate and clone identification ranged from 647 bp to 1475 bp, respectively. Clone libraries were successfully generated from swab sampling spots with high microbial counts (> 2.8 log_10_ cfu/cm^2^). No clone libraries could be generated from spots with low microbial counts (< 2.6 log_10_ cfu/cm^2^). Despite of the low TMC on the stainless steel coupons, clone libraries were successfully constructed for the coupons from December 2015 that were exposed in the milking machine for 72 h, as well as for the coupons from October 2016 and February 2017 that remained there for 48 h.

Overall, 120 isolates and 59 clone sequences from the swab samples of farm 1 and 24 isolates and 61 clone sequences from farm 2 were identified by 16S rRNA gene sequencing. Moreover, 55 isolates and 28 clone sequences from the stainless steel coupons placed in the milking machine of farm 1 were identified.

Table 2 shows the percentage distribution of the detected phyla *Actinobacteria*, *Bacteroidetes*, *Firmicutes*, and *Proteobacteria* within clone and isolate sequences of both farms. Swab samples and stainless steel coupons of farm 1 are depicted separately. Regarding the swab samples, the majority of isolate and clone sequences belonged to the phylum *Proteobacteria*, with 38% of all isolate sequences and 56% of all clone sequences of farm 1, and 42% of all isolates and 37% of all clone sequences of farm 2.

**Table 2:** Percentage distribution of phyla within isolate and clone sequences from swab samples of both farms and stainless steel coupons from farm 1.

Phylum	farm 1				farm 2	
	swab samples		coupons		swab samples	coupons
	isolate sequences	clone sequences	isolate sequences	clone sequences	isolate sequences	clone sequences
*Actinobacteria*	29%	0%	69%	28%	33%	15%
*Bacteroidetes*	7%	35%	7%	0%	8%	48%
*Firmicutes*	26%	9%	20%	18%	17%	0%
*Proteobacteria*	38%	56%	4%	54%	42%	37%

The second and third most abundant phyla were different for the culture-dependent compared to the culture-independent approach. In the isolation approach, about one third of the swab isolates of farm 1 were members of the phyla *Actinobacteria* and *Firmicutes*, respectively. However, the phylum *Actinobacteria* was not represented within the clone sequences, while the phylum *Firmicutes* was represented by only five clone sequences (9%). On farm 2, the phylum *Actinobacteria* was also the second most abundant phylum in the isolation approach with similar percentages to farm 1, followed by the phylum *Firmicutes*, to which 17% of the isolate sequences were assigned. In contrast to farm 1, 15% of the clone sequences of farm 2 belonged to the phylum *Actinobacteria*, while no clone sequence was assigned to the phylum *Firmicutes*. The phylum *Bacteroidetes*, which was represented by less than 10% of all isolate sequences of both farms, made up considerably larger percentages (35% and 48%) of the clone sequences.

The percentage distribution of phyla detected on the stainless steel coupons, which remained in the milking machine of farm 1, clearly differed from the swab samples. Most of the coupon isolates were assigned to Gram-positive phyla. The percentage of the phylum *Actinobacteria* (69%) was more than twice as high as within the swab samples, while the proportion of the phylum *Firmicutes* was similar to the swab samples. Less than 10% of the isolates from coupons were assigned to the phyla *Bacteroidetes* and *Proteobacteria*, respectively. However, more than half of the clone sequences represented the phylum *Proteobacteria*, while no *Bacteroidetes* clone sequence was obtained. In contrast to the swab samples of farm 1, about one third of the clone sequences from coupons were assigned to the phylum *Actinobacteria*, while five clone sequences from coupons (18%) represented the phylum *Firmicutes*.

Species identification as well as the number of clone and isolate sequences per sampling spot of the detected phyla *Actinobacteria*, *Bacteroidetes*, *Firmicutes* and *Proteobacteria* are presented in Table 3. Their biofilm formation, mastitis pathogenic potential described in literature, and spoilage potential is also indicated. Multiple sequences from the same farm obtained by the same approach (culture-dependent or -independent) with ≥ 99% sequence similarity were assigned to the same operational taxonomic unit (OTU). Sequences which were assigned to the same type strain or genus but with < 99% sequence similarity among each other are labelled with consecutive numbers. The Accession numbers of representative sequences for each OTU, as well as for sequences of single strains or single clones are given in Table 3.

**Table 3:** Species and genera detected in biofilm samples of farm 1 (F1) and farm 2 (F2) as determined by culture-dependent methods (isolate sequences, I) and clone libraries (C). Biofilm formation (B), mastitis pathogenic (M) and/or spoilage (S) potential are highlighted.

Genus/species	No. of sequences by isolation source							Origin		Origin of sequence	ENA Accession No.	B	M/S
	TC	R	FP	BP	EP	OB	SC	F1	F2				
***Actinobacteria***													
*Arthrobacter russicus*		1					3	I		OTU52_I	LN997877	+	L, P
*Brachybacterium* spp.			1						I	JL43	LN997883	nd	−
*Brachybacterium nesterenkovii*							1	I		M255	MK547258	nd	P
*Brachybacterium* spp.							1	I		M261	MK547259	nd	nd
*Brevibacterium celere*			1					I		M192	LN997885	+	−
*Brevibacterium casei*			1					I		M129	LN997884	+++	P
*Corynebacterium confusum*		1						I		M104	LN997888	++	−
*Corynebacterium faecale*							1	I		M299a	MK547260	nd	−
*Corynebacterium falsenii*			3					I		OTU9_I	LN997887	−	L
*Corynebacterium glutamicum*		1						I		M180a	LN997889	++	−
*Curtobacterium flaccumfaciens*		1				1		I		OTU51_I	LN997873	−	L, P
*Dermacoccus nishinomiyaensis*			1				4	I		OTU60_I	MK547261	+	L, P*
*Dietzia maris/kunjamensis*						1		I		M233	LN997892	−	L, P
*Gordonia bronchialis*						1		I		M232	LN997896	+	L
*Gordonia jacobaea/sputi*			1				1	I		OTU53_I	LN997894	−	L*
*Gordonia paraffinivorans*				1				I		M69	LN997895	++	L
*Gordonia polyisoprenivorans*			1				2	I		OTU54_I	LN997893	++	L
*Kocuria kristinae*					3		2	I		OTU6_I	LN997880	+++	L*, P*
*Kocuria palustris*						1			I	JL60	LN997879	nd	−
*Kocuria salsicia*			1		1	1	4	I		OTU10_I	LN997881	+++	L*, P*
			1		1				I	OTU7_I	LN997882	nd	L, P
*Leifsonia soli*			2				1	I		OTU11_I	LN997875	+	L*
*Microbacterium lacticum*			1				6	I		OTU55_I	LN997869	+++	P
*Microbacterium foliorum/ phyllosphaerae*		1						I		M110b	LN997868	−	−
							8	C		OTU63_C	MK547287	/	/
						2			C	OTU3_C	LN997867	/	/
*Microbacterium luteolum*			1			1			I	OTU4_I	LN997865	nd	P*
*Microbacterium maritypicum/ oxydans* 1		2	2				4	I		OTU2_I	LN998002	++	L*, P*
*Microbacterium maritypicum/ oxydans* 2						3			C	OTU1_C	LN997866	/	/
*Microbacterium testaceum*		1					1	I		OTU56_I	LN997870	−	L
*Plantibacter flavus*						1			C	C_OB2.31	LN997871	/	/
*Propioniciclava* spp.						2			C	OTU8_C	LN997886	/	/
			1						I	JL42	LN717247	nd	L
*Pseudoclavibacter changangensis*							2	I		OTU61_I	MK547262	−	−
*Pseudoclavibacter helvolus/terrae*					1			I		M230b	LN997874	−	−
*Renibacterium* spp.						1			C	C_OB2.6	LN997876	/	/
*Rhodococcus degradans*							3	I		OTU62_I	MK547263	+	L
		1							I	JL71B	LN997890	nd	−
*Rhodococcus fascians*			1				1	I		OTU57_I	LN997891	−	L
*Rothia endophytica*		2					1	I		OTU5_I	LN997878	−	P*
***Bacteroidetes***													
*Chryseobacterium* spp. 1						1			C	C_OB2.1	LN997899	/	/
*Chryseobacterium* spp. 2		1						C		C_R1.137	LN997900	/	/
*Chryseobacterium* spp. 3						1			C	C_OB2.38	LN997903	/	/
*Chryseobacterium* spp. 4		1						I		M100	LN997907	+	P
*Chryseobacterium haifense*		1						C		C_R1.99	LN997908	/	/
*Chryseobacterium bovis*							1	I		M326	MK547264	++	P, M
*Chryseobacterium carnipullorum*		2					2	I		OTU12_I	LN997897	−	−
		18						C		OTU13_C	LN997898	/	/
*Chryseobacterium chaponense*						1			C	C_OB2.8	LN997906	/	/
*Chryseobacterium ginsengiterrae*		12							C	OTU15_C	LN997909	/	/
*Chryseobacterium indoltheticum*		2							C	OTU14_C	LN997904	/	/
		1							I	JL65	LN997905	nd	P
*Chryseobacterium oncorhynchi*						1			C	C_OB2.41	LN997901	/	/
*Chryseobacterium soli*		1						I		M2	LN997902	−	L, P
*Elizabethkingia miricola*			1					I		M194	LN997910	−	−
*Empedobacter falsenii* 1		1						I		M95	LN997911	−	P
*Empedobacter falsenii* 2		1						I		M93b	LN997912	+++	−
*Flavobacterium spp*.						1			C	C_OB2.61	LN997913	/	/
*Pedobacter* spp.1						2			C	OTU16_C	LN997914	/	/
*Pedobacter* spp.2						1			C	C_OB2.51	LN997915	/	/
*Sphingobacterium* spp.1						1			C	C_OB2.16	LN997919	/	/
*Sphingobacterium* spp. 2						1			C	C_OB2.20	LN997920	/	/
*Sphingobacterium* spp.3		2							C	OTU18_C	LN997921	/	/
*Sphingobacterium cladoniae*						1			I	JL61	LN997916	nd	−
						2			C	OTU17_C	LN997917	/	/
*Sphingobacterium kitahiroshimense*						1			C	C_OB2.26	LN997922	/	/
*Sphingobacterium multivorum*							1	I		M244	MK547265	++	−
*Sphingobacterium spiritivorum*		1						I		M113	LN997918	+++	−
***Firmicutes***													
*Bacillus clausii*						1		I		M75	LN997926	−	−
*Bacillus idriensis*				1				I		M47	LN997931	++	P
*Bacillus marisflavi*		1						I		M25	LN997925	−	L
*Bacillus paralicheniformis*		2						I		OTU59_I	LN997930	+++	L
*Bacillus safensis*		2					2	I		OTU64_I	LN997932	+++	L, P
*Bacillus simplex*				1				I		M45	LN997924	−	L
		1							I	JL28	LN997923	nd	−
*Bacillus thuringiensis/ paranthracis*	1					1		I		OTU44_I	LN997934	−	L, P
*Enterococcus faecalis*					4		3	I		OTU45_I	LN997943	+	P
					2			C		OTU46_C	LN997942	/	/
*Kurthia gibsonii*						1		I		M150	LN997941	++	−
*Lactobacillus paracasei*			1		2		2	I		OTU47_I	LN997945	+	P
					2			C		OTU48_C	LN997946	/	/
*Lactobacillus fermentum*					1			I		M68a	LN997947	−	P
*Lactobacillus parabuchneri*			1					I		M164	LN997948	−	P
*Lactococcus lactis*			1						I	JL41	LN997944	nd	P
*Listeria monocytogenes*							3	C		OTU65_C	MK547288	/	M
*Macrococcus caseolyticus*						1		I		M208	LN997935	+++	P
*Paenibacillus amylolyticus*							1	I		M247	MK547266	+	P
*Paenibacillus cineris*			1					I		M137	LN997928	+	L
*Paenibacillus shunpengii*				1				I		M46	LN997927	−	−
*Pediococcus pentosaceus*		3				1		I		OTU49_I	LN997949	++	P
*Staphylococcus arleattae*							1	I		M243	MK547267	+	−
*Staphylococcus chromogenes*		1					1	I		OTU58_I	LN997937	−	L, P, M
*Staphylococcus condimenti/ carnosus/piscifermentans*				2					I	OTU50_I	LN997936	nd	P, L*
*Staphylococcus cohnii*		3						I		OTU43_I	LN997939	++	P*, L
*Staphylococcus equorum*			1					C		C_FP1.96	LN997940	/	/
*Staphylococcus haemolyticus*		1						I		M89	LN997938	−	L, M
*Staphylococcus warneri*							1	I		M258	MK547268	nd	L, P
*Staphylococcus xylosus*							2	C		OTU66_C	MK547289	/	/
***Proteobacteria***													
*Achromobacter* spp.						2			C	OTU41_C	LN997982	/	/
*Achromobacter pestifer*						3			C	OTU42_C	LN997980	/	/
						1			I	JL51	LN997981	nd	−
*Acidovorax defluvii*						1			C	C_OB2.2	LN997987	/	/
*Acinetobacter* spp.1						1			C	C_OB2.32	LN997951	/	/
*Acinetobacter* spp.2		1						C		C_R1.109	LN997953	/	/
*Acinetobacter albensis*		2					6	C		OTU32_C	LN997958	/	/
*Acinetobacter guillouiae* 1						5		C		OTU21_C	LN997954	/	/
		3	1		1	2		I		OTU30_I	LN997955	+++	L
*Acinetobacter guillouiae* 2		1							C	C_R2.8	LN997956	/	/
		1							I	JL63	LN997957	nd	−
*Acinetobacter johnsonii*		1				1		I		OTU33_I	LN997950	+++	L
*Acinetobacter parvus*		3						C		OTU34_C	LN997952	/	/
*Agrobacterium pusense*		2						I		OTU21_I	LN997998	++	−
*Brevundimonas vesicularis/nasdae*		1						C		C_R1.120	LN997992	/	/
		4						I		OTU19_I	LN997991	+++	−
*Comamonas piscis*		1							C	C_R2.27	LN997985	/	/
*Diaphorobacter* spp.		1						C		C_R1.139	LN997986	/	/
*Enterobacter hormachei*						1		I		M155	LN997969	−	P
*Escherichia coli*		1	1			1		I		OTU29_I	LN997967	+++	P*, M
*Lelliottia amnigena*						2			C	OTU37_C	LN997972	/	/
				1					I	JL34	LN997971	nd	−
*Luteibacter jiangsuensis*							1	I		M325	MK547269	−	
*Luteimonas spp*.							1	C		C_C1.17	MK547290	/	/
*Microvirgula aerodenitrificans*	1							I		M136	LN997979	+	−
*Moraxella osloensis*						1		I		M209	LN997959	−	L
*Ochrobactrum anthropi/lupini/ cytisi*				1				I		M72	MK547270	+++	−
*Ochrobactrum thiophenivorans*		1						I		M107	LN997994	++	−
*Ochrobactrum rhizosphaerae*							1	I		M331	MK547271	+	−
						1			C	C_OB2.34	LN997995	/	/
*Pandoraea pnomenusa*			2					I		OTU23_I	LN997983	+	L*
*Pantoea agglomerans*		1						I		M31	LN997968	++	−
*Paraburkholderia caledonica*							1	C		C_C1.3	MK54729	/	/
*Paracoccus laeviglucosivorans*		1						I		M177	LN997993	++	−
*Phyllobacterium* spp.						1			C	C_OB2.28	LN997996	/	/
*Pseudomonas azotoformans/ lactis*						2			I	OTU36_I	LN997965	nd	−
*Pseudomonas congelans/ syringae*						1		I		M79	LN997961	−	−
*Pseudomonas extremorientalis*						1			I	JL56	LN997963	nd	L
*Pseudomonas gessardii*						1		I		M152	LN997964	+++	−
*Pseudomonas koreensis*		1						I		M92	LN997960	+++	L, P
*Pseudomonas paralactis*							1	C		C_C1.10	MK547292	/	/
*Pseudomonas poae/trivialis*		1						I		M43	LN997962	++	L
*Raoultella terrigena/ Enterobacter aerogenes*		1						I		M93a	LN997970	+++	P
*Rheinheimera chironomi*							1	C		C_C1.6	MK547293	/	/
*Rhodanobacter glycinis*			9		6	4	5	C		OTU27_C	LN997978	/	/
*Rhizobium nepotum*		1						I		M10	LN997999	++	L
*Rhizobium radiobacter*			1						I	JL39	LN998000	nd	−
		1						I		M186	LN998001	+++	−
*Serratia marcescens*		1				1		I		OTU28_I	LN997966	+++	L*, P
*Shinella zoogloeoides*		2						I		OTU20_I	LN997997	−	−
*Sphingomonas olei*		2						I		OTU67_I	LN997989	++	L*
*Sphingobium xenophagum/ hydrophobicum*		1							C	C_R2.16	LN997990	/	/
*Stenotrophomonas lactitubi*		2						I		OTU25_I	LT222224	+++	L, P
*Stenotrophomonas maltophilia*						2		I		OTU24_I	LT222225	+++	L, P
*Stenotrophomonas rhizophila*						1			C	C_OB2.9	LN997973	/	/
		1				2			I	OTU39_I	LN997974	nd	P
*Stenotrophomonas terrae*						7			C	OTU38_C	LN997975	/	/
*Xenophilus aerolatus*		2						I		OTU22_I	LN997984	−	L

Species affiliation was determined as ≥ 98.7% sequence similarity to type strains obtained from the databases “BLAST” and “EzTaxon-e server”. One representative sequence for each species was deposited in the European Nucleotide Archive (ENA) or GenBank with the given Accession-Numbers. The number of sequences is given for the different isolation sources: teat cups (TC), milking equipment retainers (R), filter pipe (FP), stainless steel pipe at the beginning of the pressure line (BP), plastic pipe at the end of the pressure line (EP), outlet of the raw milk bulk tank (OB), and stainless steel coupon (SC). Sequences of the same farm and approach with ≥ 99% sequence similarity were assigned to the same operational taxonomic unit (OTU); sequences which were assigned to the same type strain or genus but with < 99% sequence similarity among each other are labelled with consecutive numbers. Origin of sequence: strain number, clone or representative sequence for OTU. Extent of biofilm forming ability is subdivided into weak (+), moderate (++), strong (+++), or no biofilm formation (−). Spoilage potential is subdivided in lipolysis (L) and proteolysis (P), or no spoilage potential (−). * indicates that strain specific differences of the trait were detected; “M”, considered as potential mastitis pathogen; nd, not determined; “/”, not applicable for clone sequences.

The majority of 82 isolate sequences were assigned to the phylum *Actinobacteria*. A high species richness of the genus *Microbacterium* and high numbers of isolates of the genus *Kocuria* were detected on both farms. The species *Kocuria salsicia* was isolated from different parts of the milking machine of both farms. A large proportion of the isolates assigned to the genera *Arthrobacter*, *Gordonia*, *Dermacoccus*, *Rhodococcus*, *Kocuria* and *Microbacterium* originated from the stainless steel coupons that remained in the milking machine of farm 1 for several days during ongoing operation. Except for the ladder two genera, they were not that abundant in the swab samples of the same milking machine. While nine clone sequences of farm 2 were assigned to the genera *Microbacterium*, *Plantibacter*, *Renibacterium* and *Propioniciclava*, all sequences from the coupon DNA-extracts of farm 1 were assigned to the species *Microbacterium foliorum/phyllosphaerae*.

Of the phylum *Bacteroidetes*, a high species richness of the genus *Chryseobacterium* was detected for both farms. While seven clone sequences and one isolate of farm 2 were assigned to five different OTUs of the genus *Sphingobacterium*, only two isolates of farm 1 were identified as *Sphingobacterium spiritivorum* and *Sphingobacterium multivorum*.

Only four isolates from farm 2 were designated to the genera *Bacillus*, *Staphylococcus* and *Lactococcus* of the phylum *Firmicutes*. In contrast to this, twelve isolates of farm 1 were assigned to seven different species of the genus *Bacillus*, and eight isolates and three clone sequences were assigned to seven different *Staphylococcus* species. The species *Enterococcus faecalis* and *Lactobacillus casei* were represented by clone and isolate sequences of farm 1. Three clone sequences originating from different stainless steel coupons were assigned to the species *Listeria monocytogenes*. This species was neither isolated from the swab samples nor from the stainless steel coupons of farm 1.

A high diversity of isolates and clone sequences of both farms was assigned to the phylum *Proteobacteria*. Many isolate and clone sequences belonged to different species of the genus *Acinetobacter*. Representatives of the species *Acinetobacter guillouiae* were detected on the milking equipment retainers of both farms. Different species of the genus *Pseudomonas* were isolated preferentially from the outlet of the milk bulk tank of both farms. In contrast to the genus *Acinetobacter*, to which 18 clone sequences of both farms were assigned, only one coupon clone sequence of farm 1 was assigned to the genus *Pseudomonas*. Eight isolates of different genera from the order *Enterobacterales* derived predominantly from the milking equipment retainers and the outlet of the milk bulk tank of farm 1. 24 clone sequences from various swab sampled areas as well as from the stainless steel coupons of farm 1 were designated to the species *Rhodanobacter glycinis*. This species was not represented within the isolates.

In conclusion, the culture-dependent approach led to the identification of 176 isolates from farm 1, which could be designated to 86 different species. In the parallel culture-independent approach, the identification of 85 clone sequences led to the detection of 20 different species. Six of them were also detected by the isolation approach. From the swab samples of farm 2, 24 isolates were assigned to 17 different species, while 61 clone sequences were assigned to 31 different species. Six species were detected by both approaches. In total, eight out of 135 species were detected on both farms, either by isolation or by the cloning approach.

Most of the isolates belonging to the dominating genera in the milking machine biofilms of farm 1 were classified as strong biofilm formers in microplate tests. These were the genera *Kocuria*, *Bacillus*, *Acinetobacter*, *Pseudomonas* as well as different isolates of the order *Enterobacterales* (e. g. *Serratia marcescens* and *Escherichia coli*). A high proportion of isolates with strong biofilm forming potential was found in the phylum *Proteobacteria*. While many isolates of the phylum *Actinobacteria* showed lipolytic activity, lactic acid bacteria showed proteolytic activity. Many strains of the genera *Bacillus*, *Pseudomonas*, *Serratia* and *Stenotrophomonas* were lipolytic and proteolytic.

### Statistics

The coverages of the cloning approach for the swab samples of farm 1 were 86.2% for the milking equipment retainers, 90.9% for the filter tube, and 100% for the plastic pipe at the end of the pressure line and the outlet of the milk bulk tank, respectively. The coverages for farm 2 were 84.2% for the milking equipment retainers, and 64.3% for the outlet of the bulk tank. The coverage for the stainless steel coupons placed in the milking machine of farm 1 was 85.1%. This demonstrates that most of the microbial diversity of these habitats was recovered by this analysis.

The Shannon diversity indices and Equitability values for the culture-dependent and culture-independent approaches of both farms are depicted in Table 4. The microbial diversity described by the Shannon indices was generally higher for the cultivation approaches than for the molecular approaches. As an exception, the Shannon index of the milking equipment retainers of farm 2 was higher in the cloning approach than in the cultivation approach. The highest Shannon index depicting the highest microbial diversity was detected on the stainless steel coupons and the milking equipment retainers of farm 1, while the highest diversity for farm 2 was detected on the outlet of the raw milk bulk tank.

**Table 4:** Shannon diversity index (H) and Equitability (E) values of the different sampling spots calculated for the culture-dependent isolation approach and the molecular cloning approach of farm 1 and farm 2.

	Milking equipment retainers		Filter tube		End of the pressure pipe (plastic)		Bulk tank outlet		Stainless steel Coupons	
	H	E	H	E	H	E	H	E	H	E
**Farm 1**										
Culture-dependent approach	2.35	0.98	1.39	1.0	1.33	0.83	1.56	0.87	3.22	0.94
Molecular approach	1.36	0.66	0.30	0.44	0.95	0.86	0.69	0.99	1.90	0.86
**Farm 2**										
Culture-dependent approach	1.23	1.0	1.79	1.0	0.69	1.0	3.03	0.97	/	/
Molecular approach	1.61	0.69	nc	nc	nc	nc	1.73	0.94	/	/

For farm 1, only isolates gained from the third swab sampling were included in the calculation. Nc: no clone sequences obtained, thus not calculated; “/”: no stainless steel coupons were used on farm 2.

The equal distribution of the detected diversity across the identified taxa is presented as Equitability values. These values were higher for the cultivation approaches than for the culture-independent approaches, except for the bulk tank outlets of both farms, as well as the plastic pipe at the end of the pressure line and the stainless steel coupons of farm 1, where no or only minor differences were observed. Equitability values close to 1.0 indicate a uniform distribution of taxa, whereas lower equitability values indicate the dominance of particular taxa. The latter was observed for the milking equipment retainers in the culture-independent approach of both farms as well as for the molecular approach of the filter tube of farm 1.

## Discussion

### Bacterial loads on different parts of the milking machine

The milking equipment retainers and the outlets of the milk bulk tank of both farms were spots with high microbial loads with TMCs between 3.8 and 6.3 log_10_ cfu/cm^2^. These findings were in accordance with the findings of Flach *et al*. (2014) [21], who found the highest bacterial counts on the milk drain valves of raw milk cooling tanks on dairy farms by cultivation techniques. These spots are in close contact with the cows during milking on the one hand and with the consumer and the dairy industry on the other hand. High TMCs on the milking equipment retainers could be an essential source of contamination of the milking system with biofilm formers, pathogenic bacteria and/or spoilage organisms, while high TMCs on the outlet of the bulk tank could lead to the transfer of these organisms to the dairy industry.

The high TMCs can be explained by inadequate cleaning and sanitation processes. Both spots are not covered by the CIP-procedures used to clean the milking machine after each milking. In addition, after water rinsing of the teat cups, they are placed on their retainers under wet conditions, which favors bacterial growth. Since the milking equipment retainers themselves are not cleaned at regular intervals, residues can accumulate on the retainer surface. This was confirmed by visibly loaded swabs after sampling.

The bulk tanks and their outlets are cleaned by CIP-procedures only after pickup of the raw milk by the purchaser. In case of farm 1, this takes place every three days. Within this period, farm staff frequently takes milk samples from the outlet. Additionally, during milking the bulk tank outlet is connected to the piping system of the milking machine acting as tank inlet. Despite of water rinses afterwards, milk residues may favor bacterial growth. Flach *et al*. (2014) [21] also found that the milk drain valve is difficult to clean and they observed milk residues on the swabs used for sampling.

On most sampling spots of the piping system of both farms, TMCs were lower than on the milking equipment retainers and the outlet of the bulk tank. Only the plastic pipe at the end of the pressure line of farm 1 had a TMC in the range of the bulk tank outlet. An explanation can be oxygen-limitation that might appear within the piping system and limit bacterial growth. An evidence for limited oxygen availability is the increasing abundance of lactic acid bacteria in the piping system of farm 1. Lactic acid bacteria are aerotolerant anaerobes and might have a growth advantage over obligate aerobes at low oxygen concentrations.

Except for the outlet of the milk bulk tank, the plastic surfaces of farm 1 appeared to have higher bacterial counts than the stainless steel surfaces. This could be due to the slightly higher surface hydrophobicity of plastic compared to stainless steel. Although the original surface hydrophobicity of the material plays only a minor role for bacterial adhesion, it may impact surface conditioning by milk components during milking. Modification of the physicochemical properties of the original surface may then favor bacterial adhesion and subsequent biofilm formation [40, 41]. However, such tendency was not observed on farm 2, where the plastic pipe at the end of the pressure line had a lower TMC than the stainless steel filter pipe.

Another reason for the high TMC of the plastic pipe at the end of the pressure line of farm 1 could be a higher surface roughness of the material, which could favor biofilm formation and protect bacteria from shear stress during the CIP-procedures. The plastic pipe had an operating time of several years at the moment of sampling. This contrasts with the teat cups, which are replaced at regular intervals and would argue for a higher surface roughness due to material stress. Although the effect of surface roughness on biofilm formation is adversely discussed in the literature [41], Latorre *et al*. (2010) [9] also observed that biofilms on milk meters were mainly associated with surface scratches by applying scanning electron microscopy.

The exposition of stainless steel coupons in the milking machine of farm 1 demonstrated that bacterial adhesion already took place after 48 h. The number of adhering microorganisms increased with exposition time in the milking machine.

From the relevant bacterial groups in the dairy environment, Enterobacteria and Pseudomonads were only detected on the swab sampled spots with high TMCs, i.e. the milking equipment retainers and the outlet of the milk bulk tank of both farms. In contrast to this, Gram-positive Staphylococci and LAB were the dominating groups in the piping system of farm 1, while Staphylococci were not detected in the milking machine of farm 2. These findings lead to the assumption that the usual dairy-associated microbiota dominated on spots of the milking machine with low biofilm cell density, while those taxa often associated with contamination and product spoilage predominated in biofilms with higher bacterial densities.

All swab samples were taken a few hours after the milking machines were cleaned by CIP processes. This indicates that either the procedures applied are inefficient in removing already established biofilms, or that a recontamination took place after cleaning and sanitation. This may occur, for example, if the tap water used to rinse the milking machine after the CIP procedures was contaminated with potential biofilm formers, as already discussed by Flach *et al*. (2014) [21]. However, the transfer of biofilm formers from the tap water used for rinsing the system does not account for the high diversity and cell density detected in the milking machine biofilms alone. Instead, biofilm formers can be transferred to the milking machine from various routes.

### Bacterial community composition

Both farms investigated by swab sampling had similar community compositions with respect to the detected phyla and their percentages. They also showed the same tendency towards overrepresentation of clone sequences assigned to the phylum *Bacteroidetes* and no or little representation of the phyla *Firmicutes* and *Actinobacteria*. Both approaches, cultivation and cultivation-independent, identified the phylum *Proteobacteria* as dominating part of the biofilm microbiota of swab sampled areas. In contrast to this, most bacteria (about 90%) isolated from stainless steel coupons were Gram-positive, with more than two thirds belonging to the phylum *Actinobacteria*.

Most genera of all four phyla detected in this study are frequently isolated from raw milk [18, 42] and the dairy environment [12], including biofilms in milk tankers and on dairy processing plants [8, 10, 22]. They can be considered as typical milk-associated genera.

The majority of isolates of the phylum *Actinobacteria* originated from swab samples of non-stainless steel surfaces of the milking machine of farm 1, such as the textile filter in the filter tube as well as the plastic milking equipment retainers. However, most of the stainless steel coupon isolates were assigned to this phylum. This could be a hint that early colonizers of stainless steel surfaces may differ from the bacterial community composition of mature biofilms found on this material and that the primary colonizers of the phylum *Actinobacteria* may only be present in low numbers in mature biofilms.

The detection of a high species richness of coryneform genera in milking machine biofilms and that many isolates were classified at least as weak biofilm formers in the present study confirms previous suggestions that smear cheese ripening bacteria are transferred to cheese manufacturing plants via raw milk [43, 44].

Only few isolates of the genus *Chryseobacterium* formed biofilms in microplate assays. This leads to the conclusion that members of this genus rather integrate into already established biofilms, or they may need other bacteria for biofilm formation. However, *in vitro* assays do not reflect realistic conditions in the milking machine, so that the results can only give a hint to the biofilm forming potential under *in vivo* conditions. Species of the genus *Chryseobacterium* have been isolated in the past from raw milk [45–47] as well as from beverage plant surfaces [48], which confirms their role as biofilm-associated bacteria in food production.

The detection of seven species of the genus *Bacillus* demonstrates a high species richness of this genus for farm 1. Members of this genus are known to produce heat resistant endospores, which are able to survive the pasteurization process. This favors biofilm formation on post-pasteurization pipelines in the milk-processing industry, which are then sources of recurrent contamination of the pasteurized product. Sharma & Anand (2002) [8] sampled biofilms in dairy processing plants and found that isolates of the genus *Bacillus* dominated in pre- and post-pasteurization pipelines; a similar finding was made by Malek *et al*. (2012) [49]. Not only the production of spoilage enzymes limiting shelf life and quality of milk products is of importance, but also the ability of *B*. *cereus* and relatives to produce enterotoxins, which threatens consumer health by causing food poisoning [50]. Isolates of the *B*. *cereus* group were detected on farm 1. Many of the isolates were potent biofilm, lipase and protease producers, which favors the hypothesis of transfer of biofilm formers via raw milk to the dairy industry. Although many studies have focused on *B*. *cereus* so far, other endospore forming *Bacilli* might also threaten the milk processing industry. This was confirmed by Teh *et al*. (2011) [10] who found that a *Bacillus licheniformis* isolate from a milk tanker was able to produce a heat-resistant protease and attached to stainless steel. Two isolates from the milking equipment retainers of farm 1 in this study were assigned to the closely related species *B*. *paralicheniformis*.

Seven different species of the genus *Staphylococcus* were detected on farm 1. They included the coagulase-negative species *S*. *chromogenes* and *S*. *haemolyticus*, which can cause mainly subclinical but persistent intramammary infections [51, 52]. Since they are colonizers of udder and teat skin [53], their biofilm forming ability is regarded as a potential virulence factor for causing mastitis as skin opportunists [54, 55]. In our study, Staphylocci were frequently isolated from the milking equipment retainers, which are in close contact to the udder during milking. This illustrates the potential role of the milking equipment for the transmission of mastitis pathogens within the herd.

Most of the LAB species isolated in this study have been frequently isolated from raw milk cheese, where they appear as non-starter lactic acid bacteria (NSLAB), which can be used as adjunct cultures in cheese-manufacture. NSLAB are important for the flavor-development during the ripening process of cheeses, but they are also suspected to lower product quality by producing off-flavors and other manufacturing defects [56, 57]. Since certain NSLAB species have already been detected in biofilms on dairy plants [58, 59], their detection in milking machine biofilms in this study argues for the carryover of biofilm forming organisms from dairy farms to dairy plants.

Three *Listeria monocytogenes* clone sequences originated from two different coupon DNA extracts of farm 1, which is a hint for the permanent presence of this pathogen in the milking system of farm 1. However, the presence of this organism was not confirmed by the cultivation method. Latorre *et al*. [9, 24] detected *L*. *monocytogenes* in biofilms in in-line milk filters, on milking equipment, the bulk tank outlet, and the parlor environment after selective enrichment, which was not within the scope of our study. This indicates that this pathogen is only present in low numbers in milking machine biofilms, if at all, and is unlikely to be detected by cultivation without prior enrichment.

The detection of isolates and clone sequences genetically related to the species *Acinetobacter guillouiae* and *Pseudomonas gessardii* in milking machine biofilms of both farms underlines the relevance of the genera *Acinetobacter* and *Pseudomonas* in the dairy environment. Representatives of these genera have already been described as psychrotolerant spoilage enzyme producing bacteria in the dairy environment [3, 11]. Their detection indicates that milking machine biofilms are a potential source of recurrent contamination of bulk tank raw milk with milk spoilage bacteria. Pseudomonads may also act as primary colonizers facilitating adherence and proliferation of weak or non-biofilm-formers, for example pathogens. This might also be true for the isolates detected in the milking machine biofilms of farm 1, since most of them were strong biofilm and spoilage enzyme producers.

Different species of the order *Enterobacterales* were found on both farms. *Escherichia coli* was isolated from several sampling sites of the milking machine of farm 1. Coliforms like *E*. *coli* are considered equipment hygiene indicators [60]. Their detection on the milking equipment retainers and the outlet of the milk bulk tank in combination with high bacterial counts on VRBD agar indicate insufficient sanitation efficiencies of these spots. Moreover, the detection of *E*. *coli* as a potential mastitis pathogen on the milking equipment retainers is of concern for animal health.

### Comparability of direct molecular and cultivation approaches

Most of the spots analyzed showed a lower diversity and a lower equitability for the direct cloning procedure than for the isolation approach. This means that fewer taxa were detected by the direct methods and that the detected microbiota was more frequently dominated by single taxa compared to the isolation approach. This demonstrates that results from both approaches are not congruent. The lower diversity and lower equitability of the direct method can be attributed to selecting effects of the cell lysis and PCR reactions which may lower the detection rates for cells with robust cell walls and select for 16S rRNA sequences with higher amplification efficiencies. On the other hand, some genera like *Propionibacterium*, *Epilithonimonas* and the pathogen *Listeria monocytogenes* were exclusively detected by the direct approach. This indicates that the cultivation conditions used were not appropriate for all organisms present in the samples, or that their abundance was too low to be detected without prior enrichment.

The absence of clone sequences of the phylum *Actinobacteria* in the swab samples from farm 1 and their low percentage in the coupon clone libraries cannot be explained by the low incidence of these bacteria on the sampled surfaces, since 28% of the swab isolates and 69% of the coupon isolates were assigned to this phylum in the cultivation approach. This was in accord with a similar observation during a previous analysis of the raw milk microbiota of farm 1 [18]. The high GC-content of the DNA of this phylum may lower PCR-amplification efficiency of their 16S rRNA-genes, especially in DNA-extracts of mixed bacterial communities [61, 62].

The highest Shannon indices and thus high bacterial diversities were observed for the milking equipment retainers and the stainless steel coupons of farm 1 and the outlet of the milk bulk tank of farm 2. The coverage rates of these spots indicate that the actual diversity has not yet been completely covered by analyzing the clone libraries. A tendency towards higher diversities on sampling spots with high TMCs could be observed for the swab samples of both farms. Low Equitability values were often associated with spots with low Shannon indices. This indicates a low microbial diversity accompanied by the dominance of one or few species. This was the case for the molecular approach of the milking equipment retainers of both farms as well as the filter tube of farm 1. The presented data demonstrate that the diversity of the milking system biofilm microbiota is best characterized based on a combination of isolation and cultivation-independent methods.

## Conclusions

In conclusion, the detected microbial counts and diversities reflect farm-specific differences and may have been influenced by seasonal variations. However, different genera of the order *Enterobacterales*, as well as the genera *Acinetobacter*, *Bacillus*, *Chryseobacterium*, *Kocuria*, *Microbacterium*, *Pseudomonas*, and *Staphylococcus* were detected on both farms. Most of them have already been found in raw milk, biofilms on dairy processing plants [8], and in raw milk tankers [10]. This study confirms biofilms in milking machines of dairy farms as potential source of raw milk contamination with biofilm forming bacteria. The incidence of adhering bacteria on stainless steel coupons after short time exposition in the milking machine demonstrates the speed of adherence and subsequent biofilm formation. This study is the first to describe the relevance of members of the phylum *Actinobacteria* as potential primary colonizers of stainless steel surfaces in milking machines. The difference between microbial community composition of the coupons and swab samples may reflect the shift between primary surface colonizers and the microbiota of mature biofilms.

Biofilm organisms transferred into raw milk tankers and dairy processing plants can establish new biofilms and possibly survive the pasteurization process. The secretion of spoilage enzymes from biofilms as well as the inhabitation of pathogens is a risk for milk quality and consumer health. The risk of pathogen transmission was demonstrated by the detection of *L*. *monocytogenes* sequences on the stainless steel coupon DNA extracts on different sampling occasions. This clearly indicates that the establishment of effective CIP procedures as well as a hygienically optimized plant design are essential to prevent or at least limit biofilm formation and proliferation in dairy farms. This prevention is of crucial importance to maintain a high quality of milk and its products.
